# Wortmannin Attenuates Seizure-Induced Hyperactive PI3K/Akt/mTOR Signaling, Impaired Memory, and Spine Dysmorphology in Rats

**DOI:** 10.1523/ENEURO.0354-16.2017

**Published:** 2017-06-12

**Authors:** Angela N. Carter, Heather A. Born, Amber T. Levine, An T. Dao, Amanda J. Zhao, Wai L. Lee, Anne E. Anderson

**Affiliations:** 1 The Departments of Neuroscience, Baylor College of Medicine, Houston, TX 77030; 2 Neurology, Baylor College of Medicine, Houston, TX 77030; 3 Pediatrics at Baylor College of Medicine, Houston, TX 77030; 4 The Gordon and Mary Cain Foundation Laboratories, Texas Children’s Hospital, Houston, TX 77030; 5 Jan and Dan Duncan Neurological Research Institute, Texas Children’s Hospital, Houston, TX 77030

**Keywords:** learning and memory, seizures, phosphosinositide-3 kinase, mechanistic target of rapamycin, mTOR inhibitor, wortmannin, spine morphology, protein kinase B/Akt

## Abstract

Numerous studies have shown epilepsy-associated cognitive deficits, but less is known about the effects of one single generalized seizure. Recent studies demonstrate that a single, self-limited seizure can result in memory deficits and induces hyperactive phosphoinositide 3-kinase/Akt (protein kinase B)/mechanistic target of rapamycin (PI3K/Akt/mTOR) signaling. However, the effect of a single seizure on subcellular structures such as dendritic spines and the role of aberrant PI3K/Akt/mTOR signaling in these seizure-induced changes are unclear. Using the pentylenetetrazole (PTZ) model, we induced a single generalized seizure in rats and: (1) further characterized short- and long-term hippocampal and amygdala-dependent memory deficits, (2) evaluated whether there are changes in dendritic spines, and (3) determined whether inhibiting hyperactive PI3K/Akt/mTOR signaling rescued these alterations. Using the PI3K inhibitor wortmannin (Wort), we partially rescued short- and long-term memory deficits and altered spine morphology. These studies provide evidence that pathological PI3K/Akt/mTOR signaling plays a role in seizure-induced memory deficits as well as aberrant spine morphology.

## Visual Abstract

**Figure:**
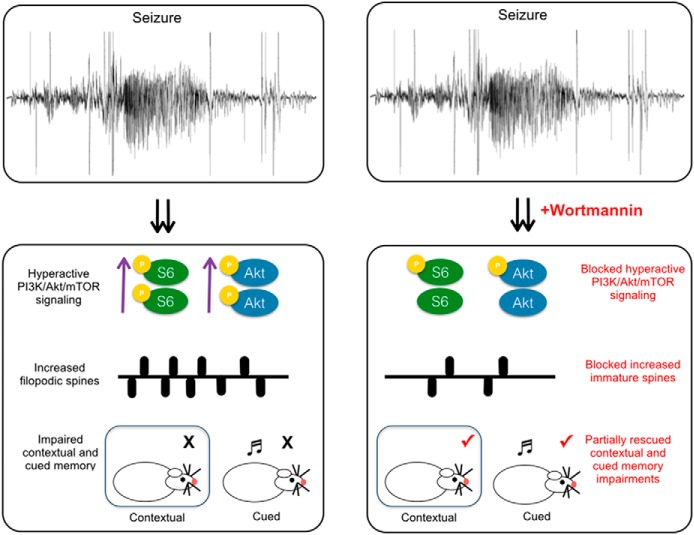

## Significance Statement

Epilepsy-associated memory deficits were originally thought to only arise in chronic epilepsy. However, the current studies demonstrate that a single generalized seizure can result not only short- but also long-term memory deficits. Furthermore, the mechanisms of how a single generalized seizure impairs memory are not well known. We find that the seizure-induced memory impairments are transient and are linked, in part, to dysregulated signaling of a memory related cascade [phosphoinositide 3-kinase/Akt (protein kinase B)/mechanistic target of rapamycin (PI3K/Akt/mTOR)] and possibly also disruptions in spine morphology, both of which are crucial for memory formation. Our studies are clinically relevant as we demonstrate that a single generalized seizure can profoundly impair memory, particularly long-term memory, despite the transient nature of the molecular and structural perturbations.

## Introduction

Seizures are characterized by transient hypersynchronous neuronal activity that is often associated with altered neuronal function (Banerjee et al., 2009; Berg et al., 2010; Fisher et al., 2014; Thom, 2014). The presence of two or more unprovoked seizures defines epilepsy, one of the most common neurologic disorders (Berg et al., 2010; Fisher et al., 2014). Along with seizures, epilepsy is also characterized by a number of comorbidities with cognitive disorders being the most prevalent (Helmstaedter et al., 2003; Nolan et al., 2004; van Rijckevorsel, 2006; Lodhi and Agrawal, 2012; Kanner, 2016). While many studies have explored how epilepsy-associated comorbidities arise, it is not clear how one self-limited generalized seizure affects cognition, in particular learning and memory. Recent studies have shown that a single generalized seizure can result in deficits in short- and long-term hippocampal-dependent memory, as well as impaired long-term amygdala-dependent memory (Mao et al., 2009; Holley and Lugo, 2016).

In addition to memory deficits, a single generalized seizure has been shown to induce hyperactivation of the phosphoinositide 3-kinase/Akt (protein kinase B)/mechanistic target of rapamycin (PI3K/Akt/mTOR) signaling cascade (Zhang and Wong, 2012). PI3K and mTOR are serine/threonine kinases that, under physiologic conditions, are necessary for learning and memory (Hoeffer and Klann, 2010). Signaling of the PI3K/Akt/mTOR pathway results in protein synthesis and cell growth (Kim et al., 2002; Sarbassov et al., 2005). In particular, PI3K/Akt/mTOR signaling has been shown to promote dendritic spine morphogenesis and remodeling (Richter and Klann, 2009; Hoeffer and Klann, 2010; Huang et al., 2013). To facilitate memory formation, dendritic spines modulate in morphology such that the spine shape converts from a long, thin immature protrusion to that of a wider and shorter mature morphology, a process that requires PI3K/Akt/mTOR signaling (Kumar et al., 2005). As such, this pathway plays a critical role in learning and memory (Kumar et al., 2005; Ehninger et al., 2008; Trinh and Klann, 2013). The fact that PI3K/Akt/mTOR signaling is dysregulated following a single generalized seizure suggests that pathologic activation of this cascade may underlie seizure-induced memory deficits. In addition, whether a single generalized seizure results in alterations in dendritic spines, and the role of PI3K/Akt/mTOR dysregulation in this process remains unclear.

In the present studies, we corroborate and extend previous findings by demonstrating that a single pentylenetetrazole (PTZ)-induced generalized seizure is associated with both short- and long-term hippocampal as well as amygdala-dependent memory deficits. Using the PI3K/Akt/mTOR inhibitor wortmannin (Wort; Brunn et al., 1996), we expand previous studies by examining the role of seizure-induced pathologic PI3K/Akt/mTOR signaling on memory deficits and dendritic spine morphology. We show, for the first time, that a single generalized seizure is associated with hippocampal dendritic spine loss and immaturity. In our pharmacological studies using Wort, we demonstrate partial rescue of the behavioral and dendritic spine changes that result from a single generalized seizure.

## Materials and Methods

### Animals

All procedures complied with and were approved by the Institutional Animal Care and Use Committee of Baylor College of Medicine and conformed to National Institutes of Health guidelines for the Care and Use of Laboratory animals. Sprague Dawley rats of either sex (Envigo) were used for biochemistry and behavioral studies. All animals were housed at the Baylor College of Medicine animal housing facilities. Animals were provided food *ad libitum* and kept on a 14/10 h light/dark cycle at 22**°**C in the Center for Comparative Medicine housing facilities. Before any experimental manipulation, all animals were handled for ∼2 min in both the induction and behavioral suites.

### Generalized seizure induction

A single generalized seizure was induced on postnatal day (P)39–P42 animals through intraperitoneal administration of the chemoconvulsant PTZ (75 mg/kg; Sigma Aldrich). Control animals received an equal volume of the vehicle saline (Sham). The age and dose were chosen based on work from previous studies as well as to improve survivability following induction (Brewster et al., 2013; Zhang and Wong, 2012) and dose-response studies (data not shown). We found that PTZ at 75 mg/kg was sufficient to result in 75% of animals exhibiting a generalized seizure with a 71% survival rate. In our hands, lower doses of PTZ resulted in <50% of animals having generalized seizures. Higher dose of PTZ were not studies due to survivability. For the molecular biology and behavioral studies, we observed the animals and recorded the seizure stages using a modified Racine scale (Racine, 1972; Lüttjohann et al., 2009). The modified Racine scale ranged from 1 to 6 and was as follows: (1) rigid posture or immobility; (2) tail clonus; (3) partial clonus with fore- or hind-limb clonus and head bobbing; (4) rearing with whole body clonus; (5) rearing and falling; and (6) tonic-clonic with loss of posture or jumping. A seizure was considered generalized when a rat exhibited stage 4 behavior corresponding to generalized seizure activity on video-electroencephalography (vEEG; see below). Any animal that did not exhibit a single, self-limited generalized convulsive seizure was not included in the behavioral testing or biochemistry studies.

### Surgery, electrode implantation, and vEEG

At P32–P35, cortical and hippocampal depth electrodes were implanted using methods described in (Sunnen et al., 2011; Brewster et al., 2013). Rats were anesthesized with isoflurane and positioned in a stereotaxic frame. Following a 1- to 2-cm midline sagittal incision, three subdural electrodes and one hippocampal-depth electrode were implanted (Plastics One). The coordinates (determined relative to Bregma) were as follows: two subdural electrodes over somatosensory cortex (1 mm posterior, 3 mm lateral) and the third electrode 4 mm posterior, ∼3 mm lateral to Bregma; the hippocampal-depth electrode was positioned in area CA1 (4 mm posterior, 2.8 mm lateral) at the depth of 2.8 mm; and the ground electrode was sutured in the cervical paraspinous region. The electrodes were held in place with Metabond (Parkell) and dental cement (Co-Oral-Ite Dental Mfg). Following a one-week recovery, animals were habituated in a recording suite and using the NicoletOne acquisition system (Natus) baseline EEG activity was recorded for 1 h. Afterward, animals received PTZ or saline as described above. The recordings were maintained continuously for 24 h thereafter (a total of 25 h). These animals were not used for any additional experimental or behavioral paradigms due to the electrode implantation.

### Pharmacological inhibition of PI3K/Akt/mTOR signaling

Wort (Selleck Chemicals) was first dissolved in dimethyl sulphoxide (DMSO; Sigma Aldrich) and then added to a vehicle solution of 1% Tween 80, 30% PEG 400. Ten minutes after seizure or saline injection, animals received either vehicle (Veh) or Wort 2.4 mg/kg intraperitoneally. We chose the Wort dose based on pilot dose-response studies (data not shown) and previously reported data (Zhang and Wong, 2012). To verify inhibition of PI3K/Akt/mTOR signaling, tissue was collected and processed for Western blotting.

### Western blotting

Separate animal cohorts were induced and whole hippocampi were collected at different time points following a single generalized seizure. Briefly, animals were deeply anesthetized using isoflurane (Piramal HealthCare) and rapidly decapitated. Hippocampi were rinsed in 1× PBS solution, frozen, and stored at −80**°**C. Tissue preparation and Western blotting on whole hippocampal tissue was performed using the methods described in (Brewster et al., 2013; Nguyen et al., 2015). Optical densities of immunoreactive bands were obtained using ImageStudioLite (LI-COR Biosciences) and normalized to GAPDH (EMD Millipore) as a loading control. Subsequently, the GAPDH-normalized bands of phosphorylated protein were normalized to GAPDH-normalized bands of total protein. Antibodies against phosphorylated (P)-Akt at T308, and Akt were used as markers of PI3K activation, while antibodies against P-S6 S240/244 and S6 were used as readouts of mTOR activation (Cell Signaling Technologies).

### Fear conditioning (FC) protocol

For all behavioral tests, the experimenters were blind to the treatment groups. One hour after generalized seizure or sham injection, animals were subjected to FC. FC was performed in sound attenuated chambers with a metal grid pan for flooring (Med Associates). Briefly, animals were placed in a novel arena and allowed to explore for 2 min. Afterward, they were subjected to two pairings of an unconditioned stimulus (US) of a 0.75-mA shock and a conditioned stimulus (CS; 72-dB white noise) with a 2 min delay between each pairing. Freezing behavior in a single cohort for the training paradigm was evaluated before further testing. Animals were then tested for short-term memory (3 h after seizure) or long-term memory (24 h after seizure). To test for contextual memory deficits, animals were tested (without the US) in the same arena in which they were trained and the time spent freezing out of a total of 5 min was scored. To test for cued memory, the animals were placed in a novel arena in a sound attenuated chamber different from the one in which they were originally trained, 1 h after contextual memory testing. After a two minute exploration period (Pre CS), the white noise was presented for the remaining 3 min (CS). Time spent freezing was collected for the Pre CS and CS conditions. Freezing, defined as the absence of any movement, was scored manually by the same experimenters for each cohort. Retention for contextual and cued memory was quantified using percentage of time freezing. To determine whether the seizure-induced memory deficits were long lasting, we performed FC training at 16 h after seizure and tested animals 24 h following training (i.e., 40 h after seizure) to screen for any long-term contextual and cued memory deficits.

### Golgi-staining and spine analyses

Golgi-Cox staining was performed according to manufacturer instructions (FD Rapid GolgiStain kit, FD Neurotechnologies). A separate cohort of animals were induced as before and 3 h after generalized seizure brains were extracted, washed with 1× PBS, and processed as per manufacturer’s instructions. Using a vibratome, hippocampal tissue sections of 150-μm thickness were collected (Leica VT 1000S). The sections were then mounted on microscope slides and dried for up to one week. Finally, the slides were dehydrated with increasing volumes of ethanol (50–100%) and coverslipped with Cytoseal 60 solution (ThermoScientific). Images of hippocampal area CA1 were obtained using a Zeiss Axio Imager M2 microscope and AxioVision software. At least 100 optical sections of 0.5-µm thickness were imaged and collated into an uncompressed z-stack image. Secondary and tertiary dendrites with lengths of at least 30 µm were selected and their spine widths and lengths traced using the protocols outlined previously (Risher et al., 2014). For each animal, four to six neurons were analyzed and 5–10 dendrites per neuron were analyzed yielding a total of 27,090 spines. Finally, statistical analyses were performed. The experimenters were blind to treatment group throughout the process of image collection, tracing, and spine analyses.

### Statistics

Prism 5 software was used for statistical analyses (GraphPad). For two group comparisons, an unpaired two-tailed Student’s *t* test with Welch’s correction to control for unequal standard deviations was used. For experiments with more than two groups, a parametric one-way ANOVA was performed. To correct for multiple comparisons, the Holm-Sidak *post hoc* test was used. When applicable, parametric two-way repeated measures ANOVA using the Holm-Sidak *post hoc* test was performed. For all tests, significance was set at *p* ≤ 0.05. A table detailing data distribution (i.e., parametric vs nonparametric distribution) has been included (Table 1). In addition, the statistical analyses, sample sizes, group effects, and *p* values for each experiment are included (Table 2).

**Table 1.** Probability analyses performed to determine data normality for each experiment

Figure/panel	Assay	Graphed residuals (Y/N)	Normality analysis	Probability of the goodness of fit (%)	Data distribution
2*A*	Training for short-term memory	No (graphed and analyzed individual scatterplot)	Nonlinear regression with Akaike's Information criterion	98.75	Parametric
2*B*	Training for long-term memory	No (graphed and analyzed individual scatterplot)	Nonlinear regression with Akaike's information criterion	99.94	Parametric
3*B*	Contextual short-term memory	Yes	Nonlinear regression with Akaike's information criterion	99.96	Parametric
3*C*, left	Cued short-term memory pre CS	Yes	Nonlinear regression with Akaike's information criterion	99.98	Parametric
3*C*, right	Cued short-term memory CS	Yes	Nonlinear regression with Akaike's information criterion	99.97	Parametric
3*D*	Contextual long-term memory	Yes	Nonlinear regression with Akaike's information criterion	99.91	Parametric
3*E*, left	Cued long-term memory pre CS	Yes	Nonlinear regression with Akaike's information criterion	99.86	Parametric
3*E*, right	Cued long-term memory CS	Yes	Nonlinear regression with Akaike's information criterion	99.96	Parametric
3*F*	Western blotting of P-S6	Yes	Nonlinear regression with Akaike's information criterion	99.97	Parametric
3*G*	Western blotting of P-Akt	Yes	Nonlinear regression with Akaike's information criterion	99.98	Parametric
4*B*	Protrusions per micrometer	Yes	Nonlinear regression with Akaike's information criterion	98.58	Parametric
4*C*	LWR	Yes	Nonlinear regression with Akaike's information criterion	99.85	Parametric
4*D*	Immature spines per micrometer	Yes	Nonlinear regression with Akaike's information criterion	98.91	Parametric
4*E*	Mature spines per micrometer	Yes	Nonlinear regression with Akaike's information criterion	86.15*	Parametric
5*B*, left	Contextual long-term memory 16-h training	Yes	Nonlinear regression with Akaike's information criterion	96.03	Parametric
5*B*, right	Cued long-term memory	No (graphed and analyzed individual scatterplot)	Nonlinear regression with Akaike's information criterion	99.94	Parametric
5*C*, right	Western blotting of P-S6 and P-Akt	Yes	Nonlinear regression with Akaike's information criterion (for S6 only),	95.53	Parametric
		Yes	Nonlinear regression with Akaike's information criterion (for AKT only)	98.17	

*Because the probability was lower than 90%, an extra sum-of-squares test was subsequently run, which reported that it is not valid to reject the null hypothesis (i.e., the data fit the model).

**Table 2.** Statistical analyses performed for each experiment

Figure/panel	Assay	Statistical test	F(df, error value), *p* value	*Post hoc* analysis/correction
2*A*	Training for short-term memory	Two-way repeated measures ANOVA	Interaction: *F*_(1,12)_ = 3.446, *p* = 0.0881Time: *F*_(1,12)_ = 42.49, *p* < 0.0001Experimental group: *F*_(1,12)_ = 1.686, *p* = 0.2185	Holm-Sidak's multiple comparisons test
2*B*	Training for long-term memory	Two-way repeated measures ANOVA	Interaction: *F*_(1,16)_ = 1.986, *p* = 0.1779Time: *F*_(1,16)_ = 88.27, *p* < 0.0001Experimental group: *F*_(1,16)_ = 0.4603, *p* = 0.5072	Holm-Sidak's multiple comparisons test
3*C*, left	Cued short-term memory pre CS	Ordinary one-way ANOVA	*F*_(3,46)_ = 16.69, *p* = 0.0100	Holm-Sidak's multiple comparisons test
3*C*, right	Cued short-term memory CS	Ordinary one-way ANOVA	*F*_(3,43)_ = 16.69, *p* < 0.0001	Holm-Sidak's multiple comparisons test
3*D*	Contextual long-term memory	Ordinary one-way ANOVA	*F*_(3,46)_ = 16.72, *p* < 0.0001	Holm-Sidak's multiple comparisons test
3*E*, left	Cued long-term memory pre CS	Ordinary one-way ANOVA	*F*_(3,46)_ = 16.69, *p* = 0.0100	Holm-Sidak's multiple comparisons test
3*E*, right	Cued long-term memory CS	Ordinary one-way ANOVA	*F*_(3,45)_ = 20.39, *p* < 0.0001	Holm-Sidak's multiple comparisons test
3*F*	Western blotting of P-S6	Ordinary one-way ANOVA	*F*_(3,13)_ = 9.391, *p* = 0.0014	Holm-Sidak's multiple comparisons test
3*G*	Western blotting of P-Akt	Ordinary one-way ANOVA	*F*_(3,11)_ = 5.058, *p* = 0.0192	Holm-Sidak's multiple comparisons test
4*B*	Protrusions per micrometer	Ordinary one-way ANOVA	*F*_(3,613)_ = 13.55, *p* < 0.0001	Holm-Sidak's multiple comparisons test
4*C*	LWR	Ordinary one-way ANOVA	*F*_(3,645)_ = 14.45, *p* = 0.0009	Holm-Sidak's multiple comparisons test
4*D*	Immature spines per micrometer	Ordinary one-way ANOVA	*F*_(3,609)_ = 4.505, *p* = 0.0039	Holm-Sidak's multiple comparisons test
4*E*	Mature spines per micrometer	Ordinary one-way ANOVA	*F*_(3,613)_ = 4.845, *p* = 0.0023	Holm-Sidak's multiple comparisons test
5*B*, left	Contextual long-term memory 16-h training	Unpaired Student's *t* test	*t*_(18.31)_ = 0.3066, *p* = 0.7626	Welch's correction
5*B*, right	Cued long-term memory	Two-way repeated measures ANOVA	Interaction: *F*_(1,21)_ = 0.5235, *p* = 0.4773Time: *F*_(1,21)_ = 133.2, *p* < 0.0001Experimental group: *F*_(1,21)_ = 0.3028, *p* = 0.5879	Holm-Sidak's multiple comparisons test
5*C*, right	Western blotting of P-S6 and P-Akt	Unpaired Student's *t* test (for S6 only)	*t*_(6.257)_ = 0.5123, *p* = 0.6260	Welch's correction
		Unpaired Student's *t* test (for AKT only)	*t*_(7.997)_ = 1.713, *p* = 0.1250	Welch's correction

## Results

### PTZ induction results in a single, self-limited generalized seizure

**Figure 1.**
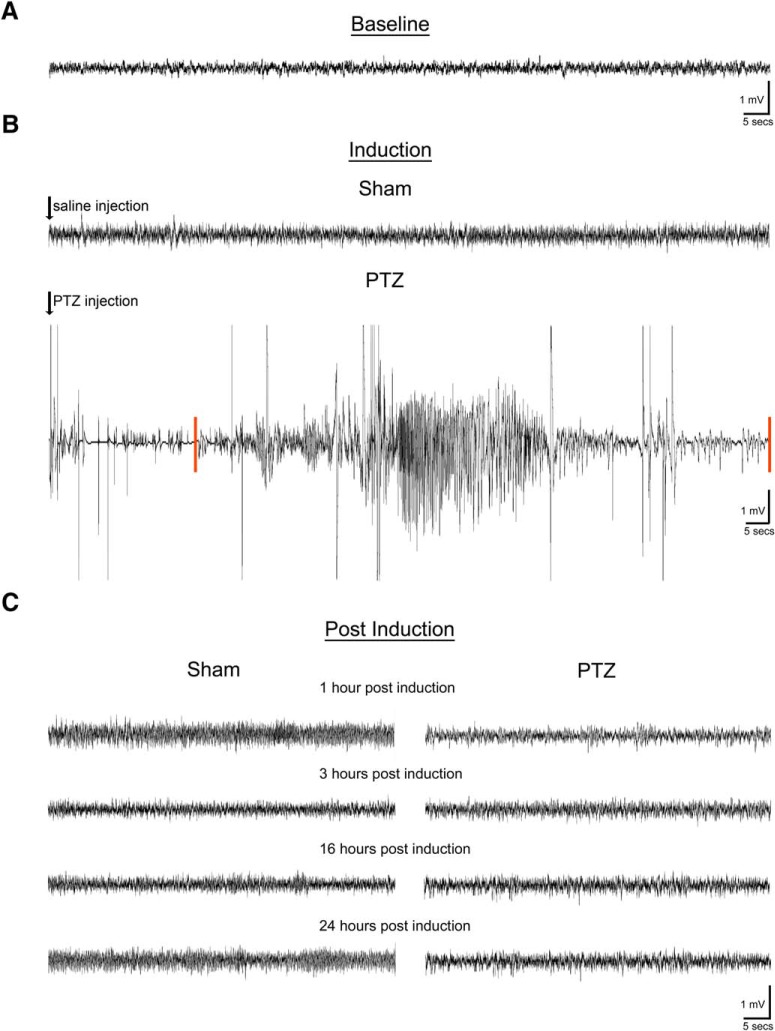
PTZ induces a single, self-limited, generalized seizure. Continuous vEEG recordings were performed in saline injected (Sham) and PTZ-induced animals for 24 h after induction. Depicted are representative hippocampal EEG traces from implanted rats before PTZ seizure induction (baseline), at the time of seizure (induction), and at time points used for behavioral training and testing. ***A***, Baseline. No overt difference in baseline EEG activity was observed. ***B***, Induction. Following saline administration (black arrow), no difference in EEG activity was observed in Sham animals (top trace). However, PTZ-seizure induction (black arrow) resulted in abnormal electrographic spikes followed immediately by a single, self-limited discharge of high-amplitude spikes observed in both the cortex (data not shown) and hippocampus (bottom trace). Red bars indicate seizure onset and termination in the hippocampus with similar findings in the cortex (data not shown). At the onset of the spike discharges (electrographic seizure onset), a behavioral Racine stage 4 generalized seizure was observed. ***C***, Postinduction. Representative EEG traces for Sham (left) and PTZ-induced animals (right) at 1, 3, 16, and 24 h after induction. There were no other seizure events in PTZ-induced animals when compared with Sham controls at any time point during the 24 h after induction (*n* = 4–5/group).

Previous studies have demonstrated that a single generalized seizure results in impaired short- and long-term hippocampal as well as amygdala-dependent memory (Mao et al., 2009; Holley and Lugo, 2016). Furthermore, hyperactive signaling of the PI3K/Akt/mTOR cascade has been shown following a single generalized seizure (Zhang and Wong, 2012). For our studies, we sought to determine whether pharmacological inhibition of the PI3K/Akt/mTOR pathway would restore seizure-induced memory deficits. First, using continuous vEEG recordings, we confirmed that a single injection of PTZ (75 mg/kg) resulted in one self-limited generalized seizure. While recordings were obtained from both the cortex and hippocampus, Figure 1 depicts representative traces from the hippocampal-depth electrode, as the PTZ injection induced electrographic seizure activity involving both the cortex and hippocampus synchronously and there was no observable difference in EEG activity between the regions. The mean latency to a generalized seizure was 8 min and 25 s with a mean duration of 56 s. There was no significant effect of animal sex on the latency to a generalized seizure.

We found that at baseline (i.e., before induction), there was no observable difference in EEG activity between PTZ-induced and Sham (PTZ vehicle injection) animals (Fig. 1*A*). Furthermore, following saline administration (black arrow), Sham animals did not exhibit any changes in behavior or EEG activity (Fig. 1*B*, top trace). However, PTZ administration led to behavioral manifestations of a generalized seizure (e.g., fore- and hind-limb clonus with rearing, Racine stage 4 and greater). vEEG recordings verified the behavioral seizure manifestations associated with high-amplitude (compared with EEG baseline activity) spike activity in the hippocampus (Fig. 1*B*, bottom trace, seizure onset and offset denoted by red bars) and in the cortex (subdural recordings; data not shown). Using continuous vEEG recordings, we confirmed that no additional seizures or epileptiform activity occurred, including the time points used for behavioral testing and training (1, 3, 16, and 24 h after induction; Fig. 1*C*). Thus, our data show that a single, self-limited, generalized seizure was induced by PTZ at 75 mg/kg.

### Inhibition of aberrant PI3K/Akt/mTOR signaling partially rescues seizure-induced memory deficits

Based on previous studies demonstrating learning and memory deficits as well as hyperactive PI3K/Akt/mTOR signaling following a single generalized seizure (Mao et al., 2009; Zhang and Wong, 2012; Holley and Lugo, 2016), we hypothesized that aberrant activation of this pathway contributes to seizure-induced memory deficits. To test our hypothesis, we determined whether pharmacological inhibition of PI3K/Akt/mTOR signaling could rescue hippocampal and amygdala-dependent memory deficits that result following a single PTZ-induced seizure. Hereafter, we refer to hippocampal and amygdala-dependent memory as contextual and cued memory, respectively.

**Figure 2.**
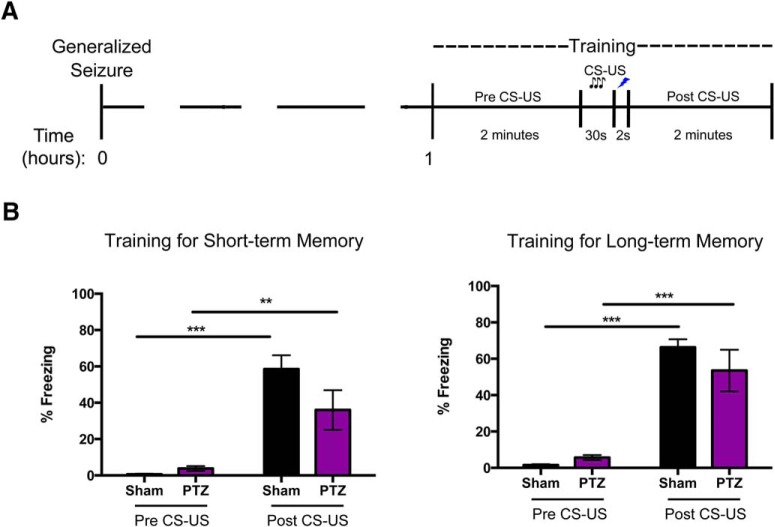
A single generalized seizure does not significantly impair FC task acquisition. ***A***, Timeline of seizure induction and the FC training protocol for short- as well as long-term memory. ***B***, Training for short-term memory (left panel). When placed in a novel arena before the presentation of the CS-US (Pre CS-US), both Sham and PTZ animals displayed a low basal level of freezing which was not significantly different between the two groups. Following the presentation of the CS (Post CS-US), both Sham and PTZ animals had a significant increase in freezing levels (*n* = 6–8/group, **p < 0.01, ***p < 0.001). Training for long-term memory (right panel). Again, before CS-US presentation, both Sham and PTZ animals displayed a low basal level of freezing, which was not significantly different between the two groups. Both Sham and PTZ animals had a significant increase in freezing levels Post CS-US (*n* = 9/group, ***p < 0.001). There was no significant difference between Sham and PTZ animals Post CS-US. Data are presented as mean ± SEM.

Before any inhibitor studies, we first verified that a single generalized seizure would not impair acquisition of the FC task. Animals were trained 1 h after seizure and assessed for freezing activity before and following the presentation of the CS (Pre CS-US and Post CS-US). In animals trained for short-term memory, we found that Pre CS-US, both PTZ and Sham animals exhibited basal levels of freezing with no significant differences between the two groups (3.77 ± 3.89% vs 0.67 ± 0.87%; Fig. 2*B*, left panel). Following the presentation of the CS (Post CS-US), there was a significant increase in the freezing levels for both PTZ (36.02 ± 30.90% vs 3.77 ± 3.89%, *p* < 0.01) and Sham (58.55 ± 18.57% vs 0.67 ± 0.87%, *p* < 0.001) animals relative to the Pre CS-US condition. However, there was no significant difference between the PTZ and Sham rats indicating that the animals can acquire the task (Fig. 2*B*, left panel). Likewise, when training the animals for long-term memory, we found no significant difference in the freezing levels of the PTZ relative to the Sham rats (5.64 ± 4.30% vs 1.54 ± 1.56%; Fig. 2*B*, right panel) in the Pre CS-US condition. Post CS-US both PTZ (53.48 ± 34.51% vs 5.64 ± 4.30%, *p* < 0.001) and Sham rats (66.28 ± 13.21% vs 1.54 ± 1.56%, *p* < 0.001) displayed significantly elevated freezing levels relative to the Pre CS-US condition. However, there was no significant difference in freezing levels between the PTZ and Sham animals Post CS-US (Fig. 2*B*, right panel). Therefore, our data show that a single, PTZ-induced seizure did not impair the responsiveness to an aversive cue as the PTZ animals display freezing behavior that is not statistically significant from Shams.

**Figure 3.**
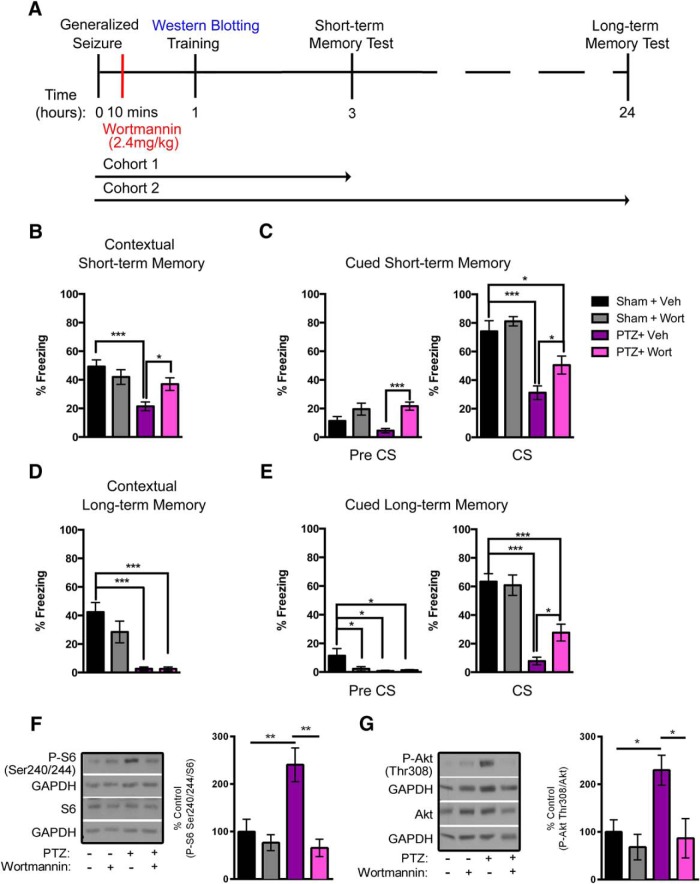
Wort partially rescues memory deficits that result from a single PTZ-induced generalized seizure. ***A***, Timeline detailing seizure induction, Wort administration, and the FC protocols. Two separate cohorts of animals were tested for short-term (cohort 1) and for long-term (cohort 2) memory. ***B***, Contextual short-term memory. PTZ + Veh animals displayed significantly reduced freezing levels relative to Sham + Veh controls (*n* = 11–12/group). Wort treatment resulted in significantly increased freezing levels in PTZ + Wort animals relative to the PTZ + Veh condition (*n* = 11–12/group). There was no significant difference between Sham + Veh and PTZ + Wort animals (*n* = 11–12/group) and between the Sham + Wort group as compared with Sham + Veh animals (*n* = 11–12/group). ***C***, Cued short-term memory. Before the CS presentation (left panel), there was no significant difference between the PTZ + Veh and Sham + Veh groups (*n* = 12–13/group). The PTZ + Wort animals displayed significantly elevated freezing levels when compared with the PTZ + Veh group (*n* = 12–13/group). There were no significant differences in freezing levels between the Sham + Veh and Sham + Wort conditions. During the CS presentation (right panel), the PTZ + Veh animals displayed significantly reduced freezing levels when compared with Sham + Veh controls (*n* = 11–12/group). While there remained a significant reduction in freezing levels in the PTZ + Wort animals compared with Sham + Veh controls (*n* = 11–12/group), PTZ + Wort animals displayed significantly increased freezing levels compared with PTZ + Veh animals (*n* = 12–13/group). There was no difference in freezing levels between Sham + Veh and Sham + Wort animals. ***D***, Contextual long-term memory. As we observed for short-term memory, there was a significant reduction in the freezing levels in PTZ + Veh animals relative to the Sham + Veh control group (*n* = 11–15/group). There was no significant difference between PTZ + Veh and PTZ + Wort animals (*n* = 11–15/group). Wort did not significantly affect freezing levels in the Sham + Wort group relative to Sham + Veh controls (*n* = 11–15/group). ***E***, Cued long-term memory. As compared with Sham + Veh controls, all other treatment groups displayed significantly reduced freezing levels in the Pre CS (left panel) condition (*n* = 11–15/group). No other significant differences were observed. During the presentation of the CS (right panel), there were significantly reduced freezing levels in PTZ + Veh animals when compared with Sham + Veh controls (*n* = 11–15/group). The PTZ + Wort group exhibited significantly reduced freezing levels as compared with the Sham + Veh group (*n* = 11–15/group). However, significantly elevated freezing levels were present in PTZ + Wort animals relative to PTZ + Veh group (*n* = 11–15/group). Wort treatment did not affect the freezing levels in Sham animals. Representative Western blottings (left panels) and quantifications (right panels) depicting P-S6 (***F***) and P-Akt (***G***) immunoreactivity from whole hippocampal homogenates of animals from all treatment groups 1 h following induction are shown. ***F***, There were significantly elevated P-S6 levels in the PTZ + Veh animals relative to Sham + Veh controls (*n* = 3–6/group). The PTZ + Wort group displayed significantly reduced P-S6 levels as compared with PTZ + Veh animals (*n* = 3–6/group). P-S6 levels were not significantly changed in Sham + Wort compared with Sham + Veh animals (*n* = 3–6/group). ***G***, Similarly, in PTZ + Veh animals, P-Akt levels were significantly elevated relative to Sham + Veh animals (*n* = 3–6/group). The P-Akt levels were reduced in PTZ + Wort animals the relative to PTZ + Veh group (*n* = 3–6/group). There was no significant difference in P-Akt levels between Sham + Wort and Sham + Veh animals (*n* = 3–6/group). All data are represented as mean ± SEM (*p < 0.05, **p < 0.01, ***p < 0.001).

Next, we determined whether pharmacological inhibition of the PI3K/Akt/mTOR cascade would rescue memory deficits following a single generalized seizure. Ten minutes after a PTZ-induced generalized seizure, Sham and PTZ animals received either vehicle (Veh) or the PI3K inhibitor Wort. One cohort of animals was tested for short-term contextual and cued memory 3 h after induction (cohort 1; Fig. 3*A*). A second cohort of animals were induced and trained as above, but tested at 24 h after seizure for long-term contextual and cued memory (cohort 2; Fig. 3*A*).

We found that, following a single generalized seizure, the animals displayed impairments in short-term contextual memory. Freezing time was significantly reduced in PTZ + Veh relative to Sham + Veh animals (21.40 ± 11.16% vs 49.28 ± 15.47%, respectively, *p* < 0.001; Fig. 3*B*). Wort was sufficient to rescue the seizure-induced reduction in freezing levels in PTZ + Wort compared with PTZ + Veh animals (36.94 ± 15.32% vs 21.40 ± 11.16%, respectively, *p* < 0.05). There were no significant differences in the freezing levels in Sham + Wort animals relative to Sham + Veh controls (41.96 ± 17.05% vs 49.28 ± 15.47%, respectively; Fig. 3*B*).

We assessed whether blocking seizure-induced hyperactive PI3K/Akt/mTOR signaling with Wort treatment rescued cued memory deficits. When placed in a novel context, rats from all of the treatment groups exhibited basal freezing levels. In the Pre CS condition, there was no significant difference in the percentage freezing in PTZ + Veh relative to Sham + Veh animals (4.54 ± 5.60% vs 11.36 ± 9.99%, respectively, Fig. 3*C*, left panel). However, there was a significant reduction in freezing levels in PTZ + Veh relative to PTZ + Wort rats (4.54 ± 5.60% vs 21.67 ± 9.77%, respectively, *p* < 0.001; Fig. 3*C*, left panel) before cue presentation. There was no significant difference in the percentage freezing between Sham + Veh (11.36 ± 9.99%), Sham + Wort (19.57 ± 13.85%) and PTZ + Wort animals (21.67 ± 9.77%) (Fig. 3*C*). Once the cue (CS) was presented, all experimental groups displayed elevated freezing levels relative to the Pre CS condition. However, there was a significant reduction in the percentage of time freezing in PTZ + Veh relative to Sham + Veh animals (31.18 ± 17.24% vs 74.11 ± 24.86%, respectively, *p* < 0.001; Fig. 3*C*). Wort treatment was not sufficient to restore the significantly reduced freezing levels in PTZ + Wort relative to the Sham + Veh animals (50.54 ± 21.72% vs 74.11 ± 24.86%, respectively, *p* < 0.05; Fig. 3*C*). In contrast, there was a significant increase in the percentage of time freezing in PTZ + Wort animals relative to the PTZ + Veh group (50.54 ± 21.72% vs 31.18 ± 17.24%, respectively, *p* < 0.05; Fig. 3*C*) consistent with a partial rescue. Finally, there were no significant differences in freezing levels in Sham + Wort relative to Sham + Veh rats (81.15 ± 10.89% vs 74.11 ± 24.86%, respectively; Fig. 3*C*, right panel). These data suggest that by blocking the seizure-induced elevation in PI3K/Akt/mTOR signaling, we were able to partially rescue deficits in short-term contextual and cued memory.

We also evaluated whether blocking elevated PI3K/Akt/mTOR signaling could rescue seizure-induced deficits in long-term contextual memory. Similar to that observed in the short-term memory test, there were significantly decreased freezing levels in PTZ + Veh relative to Sham + Veh controls (2.61 ± 4.36% vs 42.40 ± 22.21%, respectively, *p* < 0.001; Fig. 3*D*) in the long-term contextual memory test. However, unlike in the short-term contextual memory test, there was a significant reduction in freezing levels in PTZ + Wort compared with Sham + Veh animals (2.53 ± 5.23% vs 42.40 ± 22.21%, respectively, *p* < 0.001; Fig. 3*D*). There was no significant difference between the Sham + Veh and Sham + Wort groups (42.40 ± 22.21% vs 28.44 ± 26.22%, respectively; Fig. 3*D*).

As we observed in the short-term cued memory test, there was a partial rescue with Wort treatment following a single generalized seizure in the long-term cued memory test. We first evaluated the percentage of time freezing for animals placed in a novel environment before cue presentation. All Pre CS treatment groups exhibited basal levels of freezing, with the PTZ + Veh (0.75 ± 1.63%), PTZ + Wort (1.32 ± 1.67%), and Sham + Wort (2.22 ± 5.27%) having significantly reduced freezing levels relative to Sham + Veh animals (11.40 ± 16.64%, *p* < 0.05; Fig. 3*E*). As we observed in the short-term cued memory test, the Pre CS freezing levels for all treatment groups were lower than the CS condition. On CS presentation, there were significantly lower freezing levels in the PTZ + Veh compared with Sham + Veh rats (7.82 ± 9.10% vs 63.38 ± 18.43%, respectively, *p* < 0.001; Fig. 3*E*, right panel). Comparable to what we observed in the short-term cued memory test, PTZ + Wort animals displayed significantly lower freezing levels compared with Sham + Veh (*p* < 0.001), but the freezing levels were significantly higher in PTZ + Wort compared with PTZ + Veh rats (27.72 ± 22.89% vs 7.82 ± 9.10%, respectively, *p* < 0.05; Fig. 3*E*), suggesting a partial rescue. Additionally, there was no significant differences in the freezing levels between the Sham + Veh and the Sham + Wort groups (63.38 ± 18.43% vs 60.47 ± 24.76%, respectively; Fig. 3*E*) in the CS condition of the long-term cued memory test. These results suggest that by blocking PI3K/Akt/mTOR signaling following a single generalized seizure, we were able to partially rescue long-term contextual and cued memory deficits.

To verify that Wort effectively blocked the seizure-induced dysregulation of PI3K/Akt/mTOR signaling, we performed Western blotting of downstream effectors of the PI3K/Akt/mTOR pathway in hippocampal homogenates 1 h following seizure induction corresponding to the time at which the animals were trained (Fig. 3*F*,*G*). For these studies, we used antibodies that recognize P-S6 at S240/244 and S6 as well as P-Akt at T308 and Akt. The levels of P-S6 and P-Akt were used as readouts of PI3K/Akt/mTOR activation. Our analyses revealed a significant increase in the P-S6 levels in PTZ + Veh as compared with Sham + Veh rats (240.50 ± 70.65% vs 100 ± 58.44%, respectively, *p* < 0.01; Fig. 3*F*) 1 h after seizure. There was a significant reduction of P-S6 levels in PTZ + Wort compared with PTZ + Veh animals (65.80 ± 41.12 vs 240.50 ± 70.65, respectively, *p* < 0.01; Fig. 3*F*). There was no significant difference in the P-S6 levels in Sham + Veh compared with Sham + Wort animals (100 ± 58.44% vs 76.67 ± 29.26%, respectively; Fig. 3*F*).

Similar results were found with P-Akt. Following a single generalized seizure, there was a significant increase of P-Akt levels in PTZ + Veh animals relative to Sham + Veh controls (229.50 ± 63.32% vs 100 ± 50.44%, respectively, *p* < 0.05; Fig. 3*G*). P-Akt levels were significantly reduced in PTZ + Wort relative to PTZ + Veh rats (86.75 ± 82.84% vs 229.50 ± 63.32%, respectively, *p* < 0.05; Fig. 3*G*). There was no significant difference in the P-Akt levels in Sham + Veh compared with Sham + Wort animals (100 ± 50.44% vs 68.33 ± 46.52%, respectively; Fig. 3*G*). Taken together, these data indicate that a single injection of Wort was sufficient to block seizure-induced increases in PI3K/Akt/mTOR signaling. Furthermore, by inhibiting activation of this cascade, we were able to partially rescue the seizure-induced short and long-term memory deficits.

### Inhibition of aberrant PI3K/Akt/mTOR signaling rescues seizure-induced aberrant spine morphology but not spine loss in hippocampal CA1

Previous studies have reported altered dendritic structure and decreases in spine number following prolonged seizure activity (or status epilepticus; Ouyang et al., 2007; Zeng et al., 2007). However, it is unclear how dendritic spine morphology is affected following a single brief and self-limited generalized seizure. Given the important role of spine morphology in learning and memory, and the known role of the PI3K/Akt/mTOR pathway in regulating spine structure, we evaluated whether there were seizure-induced spine changes and whether seizure-induced hyperactive PI3K/Akt/mTOR signaling is associated with these changes.

**Figure 4.**
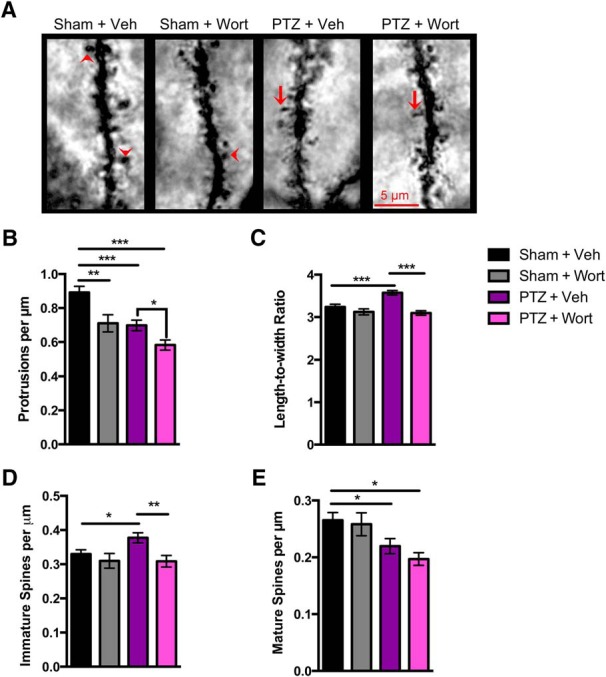
A single PTZ-induced seizure leads to dendritic spine alterations in hippocampal dendrites with partial rescue using Wort. Brains were collected 3 h following a single generalized seizure and images of hippocampal area CA1 secondary and tertiary dendrites were obtained. ***A***, High-magnification representative photomicrographs of dendrites in Sham and PTZ animals treated either with Veh or Wort. Mature spines are observed in Sham + Veh and Sham + Wort animals (red arrowheads), while dendritic spines from animals in the PTZ + Veh and PTZ + Wort conditions display immature shaped spines (red arrows). Wort treatment reduced the number of visible spines in both Sham and PTZ animals. Less immature spines are observed in PTZ + Wort animals. ***B***, Analyses revealed that a single generalized seizure resulted in a significant reduction of the number of spine protrusions per micrometer relative to Sham + Veh animals (*n* = 6 animals/group, *n* = 4–6 neurons/animal, *n* = 94–113 dendrites/group). In the PTZ + Wort condition, the number of protrusions per micrometer was significantly reduced relative to Sham + Veh controls and further reduced relative to PTZ + Veh animals (*n* = 3–6 animals/group, *n* = 4–6 neurons/animal, *n* = 113–135 dendrites/group). There was also a significant reduction in Sham + Wort relative to Sham + Veh (*n* = 3 animals/group, *n* = 4–6 neurons/animal, *n* = 94 dendrites/group). ***C***, In PTZ + Veh animals, there was a significant increase in the LWR relative to Sham + Veh controls of those spines that remained (*n* = 6 animals/group, *n* = 4–6 neurons/animal, *n* = 94–113 dendrites/group). The LWR was significantly reduced in PTZ + Wort animals as compared with the PTZ + Veh condition (*n* = 3–6 animals/group, *n* = 4–6 neurons/animal, *n* = 113–135 dendrites/group). No significant differences in LWR were found between Sham + Wort and Sham + Veh animals (*n* = 3–6 animals/group, *n* = 4–6 neurons/animal, *n* = 113–135 dendrites/group). ***D***, Further analyses of spine morphology revealed that in the PTZ + Veh experimental group, there was a significant increase in the number of immature spines per micrometer relative to Sham + Veh animals (*n* = 6 animals/group, *n* = 4–6 neurons/animal). Wort treatment blocked the seizure-induced increase in immature spines per micrometer in PTZ + Wort when compared with PTZ + Veh condition (*n* = 3–6 animals/group, *n* = 4–6 neurons/animal, *n* = 113–135 dendrites/group). In Sham + Wort animals, there was no significant difference in the number of immature spines per micrometer relative to Sham + Veh animals (*n* = 6 animals/group, *n* = 4–6 neurons/animal). ***E***, Finally, in PTZ + Veh animals, there was a significant reduction in the number of mature spines per micrometer when compared with Sham + Veh controls (*n* = 6 animals/group, *n* = 4–6 neurons/animal, *n* = 94–113 dendrites/group). There was no significant difference in the number of mature spines per micrometer in PTZ + Wort as compared with PTZ + Veh animals indicating Wort did not block the seizure-induced decrease in mature spines (*n* = 3–6 animals/group, *n* = 4–6 neurons/animal, *n* = 94–135 dendrites/group). There was no effect of Wort on the number of mature spines per micrometer in Sham + Wort compared with Sham + Veh animals (*n* = 3–6 animals/group, *n* = 4–6 neurons/animal, *n* = 94–135 dendrites/group). Data represented as mean ± SEM (*p < 0.05, **p < 0.01, ***p < 0.001).

We determined whether a single generalized seizure was sufficient to alter the number of dendritic spines. In the current studies, we focused our analyses on the secondary and tertiary dendrites in stratum radiatum of hippocampal area CA1. There was a significant reduction the number of dendritic spine protrusions in PTZ + Veh relative to Sham + Veh animals (0.70 ± 0.44 vs 0.89 ± 0.50 spines/µm, respectively, *p* < 0.001; Fig. 4*B*). A further decrease in the number of dendritic spines was observed in the PTZ + Wort relative to PTZ + Veh rats (0.58 ± 0.34 vs 0.70 ± 0.44 spines/µm, respectively, *p* < 0.05; Fig. 4*B*). There was also a significant reduction in the number of dendritic spine protrusions in Sham + Wort relative to the Sham + Veh animals (0.71 ± 0.5 vs 0.89 ± 0.50 spines/µm, respectively, *p* < 0.01; Fig. 4*B*), suggesting that inhibition of PI3K/Akt/mTOR signaling leads to a decrease in spine number in Sham animals. Furthermore, a single self-limited generalized seizure also resulted in a decreased spine number, which could not be rescued by blocking hyperactive PI3K/Akt/mTOR signaling.

To ascertain how a single generalized seizure affects spine morphology, we traced the length and width of dendritic spines to obtain spine length-to-width ratios (LWRs) for animals in each experimental group. A single generalized seizure resulted in a significant increase in the LWR in the dendritic spines of PTZ + Veh relative to Sham + Veh animals (3.60 ± 0.83% vs 3.20 ± 0.82%, respectively, *p* < 0.001; Fig. 4*C*). Blocking the seizure-induced increase in PI3K/Akt/mTOR signaling using Wort resulted in a significant reduction in the LWR in PTZ + Wort relative to PTZ + Veh animals (3.10 ± 0.62% vs 3.60 ± 0.83%, respectively, *p* < 0.001), but had no significant effect on Sham + Wort relative to Sham + Veh controls (3.10 ± 0.70% vs 3.20 ± 0.82%, respectively; Fig. 4*C*). Because we observed a significant increase in the LWR following a single generalized seizure, we hypothesized that there would be an increase in long, immature spine types. As such, we categorized and quantified dendritic spines of differing morphologies and determined whether inhibiting hyperactive PI3K/Akt/mTOR signaling was sufficient to block these changes. The analyses demonstrated a significant increase in the number of immature spines (red arrows) in PTZ + Veh compared with Sham + Veh animals (0.38 ± 0.21 vs 0.33 ± 0.17 immature spines/µm, respectively, *p* < 0.05; Fig. 4*A*,*D*). There was a significant reduction in the number of immature spines in PTZ + Wort compared with PTZ + Veh animals (0.31 ± 0.20 vs 0.38 ± 0.21 immature spines/µm, respectively, *p* < 0.01). There was no significant difference in the number of immature spines in Sham + Wort relative to Sham + Veh animals (0.31 ± 0.21 vs 0.33 ± 0.17 immature spines/µm respectively; Fig. 4*A*,*D*). Thus, a single generalized seizure was associated with an increase in immature, or immature dendritic spines, and by inhibiting PI3K/Akt/mTOR hyperactivity, we were able to block this seizure-induced increase.

Next, we examined whether the number of mature dendritic spines (red arrowheads), were altered following a single generalized seizure and effect of PI3K/Akt/mTOR blockade on this spine type. Indeed, we found a significant reduction in the number of mature spines in PTZ + Veh animals relative to the Sham + Veh group (0.22 ± 0.19 vs 0.27 ± 0.19 mature spines/µm, respectively, *p* < 0.05; Fig. 4*A*,*E*). The number of mature spines remained reduced in PTZ + Wort compared with the Sham + Veh animals (0.20 ± 0.13 vs 0.27 ± 0.19 mature spines/µm, respectively, *p* < 0.05). Finally, our analyses revealed no difference in the number of mature spines in Sham + Wort relative to the Sham + Veh rats (0.26 ± 0.20 vs 0.27 ± 0.19 mature spines/µm, respectively; Fig. 4*A*,*E*). Thus, in parallel with an increase in immature spines, there was a decrease in mature spines following a single generalized seizure. In addition, while PI3K/Akt/mTOR inhibition was sufficient to block the increase in immature spine protrusions, the concurrent decrease in mature spines was not rescued.

### Seizure-induced memory deficits and altered PI3K/Akt/mTOR signaling are transient

**Figure 5.**
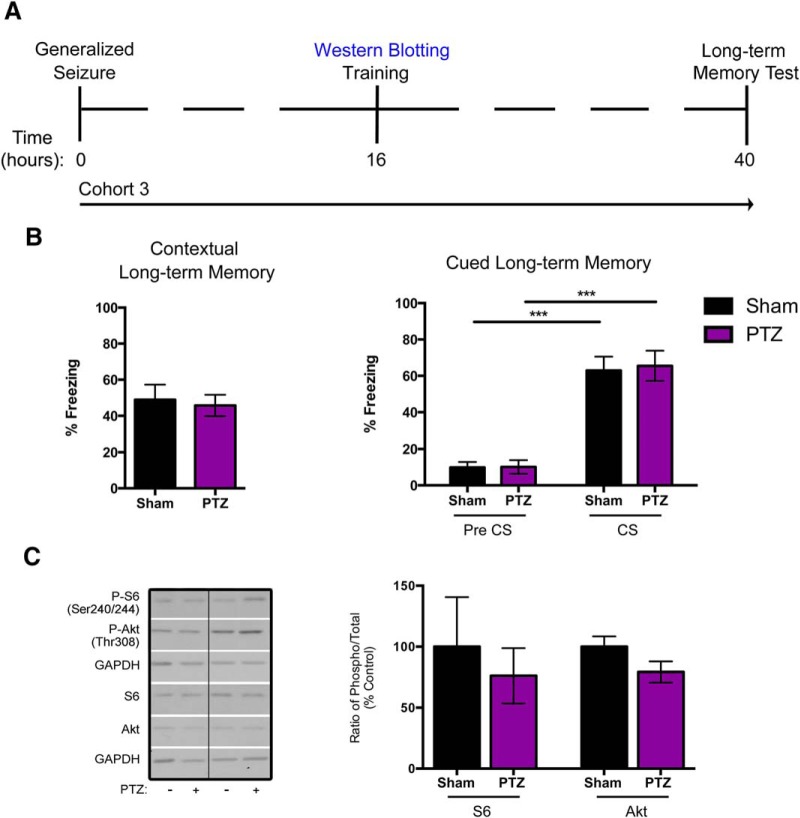
Seizure-induced long-term contextual and cued memory deficits and PI3K/Akt/mTOR hyperactivation are transient. ***A***, Timeline of seizure induction and FC protocol is shown. A third group of animals (cohort 3) was induced as before but trained 16 h after seizure in FC. Twenty-four hours after the training (40 h after seizure), the animals were tested for long-term contextual and cued memory. ***B***, Contextual long-term memory (left panel). When tested at 40 h after seizure, both Sham and PTZ animals displayed elevated freezing levels with no significant difference between groups (*n* = 11–12/group). Cued long-term memory (right panel). There was no significant difference between the Sham and PTZ animals in the Pre CS condition (*n* = 11–12/group). During the CS presentation, both Sham and PTZ animals displayed significantly elevated freezing levels relative to the Pre CS condition, and there was no significant difference between the two treatment groups (*n* = 11–12/group). ***C***, Representative Western blotting from whole hippocampi and quantifications of P-S6 and P-Akt (left and right blot panels, respectively) at 16 h following a single seizure are shown. The bar graphs from the analysis reveal that at 16 h following a single generalized seizure there was no significant difference in the P-S6 and P-Akt levels in PTZ animals relative to Sham controls (*n* = 5/group). Data represented as mean ± SEM (*p < 0.05, **p < 0.01, ***p < 0.001).

We evaluated whether long-term memory deficits following a single generalized seizure are transient. Seizures were induced in the rats as described before (cohort 3) but the animals were trained 16 h after seizure and tested 24 h after training for long-term contextual and cued memory (40 h after seizure; Fig. 5*A*). We found that in contextual long-term memory, there was no significant difference in the percentage of time freezing between Sham and PTZ groups (48.88 ± 27.95% vs 45.72 ± 20.59%, respectively; Fig. 5*B*, left panel). Similarly, when animals were tested for cued long-term memory, our analyses showed no significant differences in the percentage freezing during either the Pre CS condition in Sham compared with PTZ rats (10.79 ± 10.30% vs 10.98 ± 13.86%, respectively) or in the CS condition (63.66 ± 8.27% vs 70.92 ± 6.81%, respectively; Fig. 5*B*). Thus, by this time point there was recovery of the long-term memory deficits induced by a single generalized seizure, suggesting that these alterations were transient.

We assessed PI3K/Akt/mTOR signaling at 16 h following a single generalized seizure (training time point for cohort 3) and found that it had returned to basal levels (Fig. 5*C*). At 16 h after seizure, there was no significant difference in P-S6 levels between Sham and PTZ animals (100 ± 40.72% vs 76.13 ± 22.64%, respectively), nor was there a significant difference in the P-Akt levels in Sham compared with PTZ rats (100 ± 8.45% vs 79.26 ± 8.65%, respectively; Fig. 5*C*, right panel). These data suggest that both the memory deficits and hyperactive signaling of PI3K/Akt/mTOR following a single generalized seizure are transient and resolve within 16 h of the seizure.

## Discussion

In the present study, we demonstrated that a single generalized seizure resulted in contextual and cued memory deficits, dysregulated PI3K/Akt/mTOR signaling, and altered dendritic spine morphology. Furthermore, we showed that by inhibiting seizure-induced hyperactivation of PI3K/Akt/mTOR signaling, we were able to rescue short-term contextual memory, partially rescue short- and long-term cued memory, and block the increase in hippocampal immature spines induced by a single generalized seizure. Inhibition of PI3K/Akt/mTOR signaling did not restore the seizure-induced long-term contextual memory deficits or the decrease in mature spines. We found that these changes were transient as rats that underwent a single generalized seizure had no significant differences in long-term cued or contextual memory compared with Shams when trained 16 h following a single generalized seizure, at which point PI3K/Akt/mTOR signaling was no longer hyperactivated. These findings suggest that dysregulation of the PI3K/Akt/mTOR pathway and aberrant spine morphology may contribute to seizure-induced memory deficits. Furthermore, there is a window of time during which memory is disrupted following a single generalized seizure, with later recovery, which has potential clinical relevance.

Our findings using rats are consistent with and extend previous work. Compared with previous studies using the single generalized seizure model, Mao et al. (2009) reported impaired short- and long-term contextual fear memory in rats. However, they did not test contextual learning. Holley and Lugo (2016) reported that a single generalized seizure in mice resulted in deficits in long-term cued memory, had no effect on locomotion, and did not impair cued fear acquisition.

In epilepsy models, where animals have multiple spontaneous seizures, a significant decrease in the number of spine protrusions in hippocampal dendrites has been recorded (Jiang et al., 1998; Zha et al., 2005; Zeng et al., 2007; Guo et al., 2012; Brewster et al., 2013; Nie et al., 2015). In the current studies, we demonstrate that a single, self-limited generalized seizure is sufficient to reduce spine number. To our knowledge, this has not previously been shown. Moreover, we found that of the spines that remained, there was a significant increase in immature and a concurrent decrease in mature spine types. A significant increase in the number of immature spines has been evident in chronic epilepsy where cognitive impairments are described and in genetic models of cognitive disorders such as Fragile X syndrome, autism, and tuberous sclerosis (Penzes et al., 2011; Pathania et al., 2014; Kim et al., 2016).

Other studies using epilepsy models report altered structure of the dendritic shaft and decreases in the number of dendritic branches (Jiang et al., 1998; Ouyang et al., 2007; Zeng et al., 2007; Guo et al., 2012; Brewster et al., 2013). Using the single seizure model, we did not observe any overt morphologic changes in the dendrites themselves based on qualitative visual inspection (data not shown). The observed memory deficits following a single generalized seizure may be, at least in part, due to shifts in spine morphology (maturity) and number. If this is the case, we expect these changes to be transient.

Hyperactive PI3K/Akt/mTOR signaling has been well characterized in epilepsy-associated memory deficits (Ehninger et al., 2008; Brewster et al., 2013; Cambiaghi et al., 2013; Trinh and Klann, 2013; Lugo et al., 2014). Recently, Zhang and Wong (2012) reported that a single generalized seizure was sufficient to cause dysregulated signaling of PI3K/Akt/mTOR pathway. In the current studies, we show that by blocking seizure-induced hyperactive PI3K/Akt/mTOR signaling at the time of training, we are able to rescue deficits in hippocampal-dependent and partially rescue amygdala-dependent memory. We were able to block hyperactive PI3K/Akt/mTOR signaling following a single generalized seizure. A previous study demonstrated that pathway inhibition before seizure induction also suppressed PI3K/Akt/mTOR pathway activation (Zhang and Wong, 2012). In the current studies, dysregulated PI3K/Akt/mTOR activity appears to partially contribute to seizure-induced deficits in hippocampal and amygdala-dependent memory. When we blocked hyperactive PI3K/Akt/mTOR signaling following a single generalized seizure, we partially rescued both cued short- and long-term memory. Thus, our results suggest that amygdala-dependent memory deficits may only partially be attributed to dysregulation of the PI3K/Akt/mTOR pathway. In contrast, PI3K/Akt/mTOR inhibition following a single generalized seizure rescued only short-term contextual memory suggesting that with hippocampal-dependent memory, there are additional mechanisms that contribute to the long-term memory deficits that are possibly related to the conversion from short to long-term memory.

Pathologic PI3K/Akt/mTOR pathway activation seems to correlate with the seizure-induced memory deficits because 16 h after seizure, when the PI3K/Akt/mTOR pathway is no longer activated, the deficits in contextual and cued memory were not observed. Additional future studies are needed to further evaluate the role of PI3K/Akt/mTOR dysregulation and other molecular mechanisms underlying seizure-induced memory deficits and dendritic changes.

Since results from our lab and others demonstrate that a single generalized seizure results in aberrant PI3K/Akt/mTOR signaling, it is conceivable that excessive protein translation may underlie the observed memory deficits. However, there are recent reports that activation of mTOR downstream targets may not lead to increased translation (Biever et al., 2015). Akt signaling has been reported to regulate actin polymerization, spine number, and subsequently, synaptic potentiation and memory (Huang et al., 2013). Moreover, Kumar et al., found that constitutive activation of Akt signaling results in increased expression of filopodic spines in cultured hippocampal neurons (Kumar et al., 2005). Based on these studies and our findings showing that inhibiting the seizure-induced elevation in PI3K/Akt/mTOR signaling blocked the seizure-induced increase in immature spine types, it is possible that these changes in spine morphology are, at least in part, the result of aberrant PI3K-Akt signaling.

In summary, our data show that a single generalized seizure and the associated memory impairments are linked, in part, to dysregulated signaling of PI3K/Akt/mTOR cascade and altered spine morphology and that these changes are transient. Based on these findings and those from other labs, there is a window of time following a single generalized seizure in rodents during which there are significant short- and long-term memory deficits with associated disruption of PI3K/Akt/mTOR signaling and dendritic spines, both of which are important determinants of memory. These studies have translational relevance in clinical epilepsy as these findings indicate that a single generalized seizure significantly impacts memory for up to 24 h.

## Synthesis

Reviewing Editor: Jorge J. Palop, Gladstone Institutes and UCSF Decisions are customarily a result of the Reviewing Editor and the peer reviewers coming together and discussing their recommendations until a consensus is reached. When revisions are invited, a fact-based synthesis statement explaining their decision and outlining what is needed to prepare a revision will be listed below.

SYNTHESIS:

The manuscript advances the field by demonstrating that a single generalized seizure results in both contextual and cued fear conditioning memory deficits. In addition, the manuscript shows that inhibition of the hyperactive PI3K/AKT/mTOR signaling pathway attenuates PTZ-induced short-term contextual memory deficits. However, there are several major concerns that need to be resolved, including proper description of the statistical methods and the potential confound of hyperactivity in the fear conditioning data.

Major comments:

1) The statistical methods need to be described in detail for each experiment, including information regarding the specific statistical test performed (e.g., ANOVA, post hoc test, t-test, etc.), n numbers, and mouse groups. This information needs to be clearly defined in the result section or legends. ANOVA and post hoc for multiple comparisons need to be performed and described correctly. The results cannot be properly evaluated without this information.

2) The methods/results need to address if there was a sex effect in the outcome measures since both sexes were used, describe the methods used to quantify the severity and number of seizures (Racine scale? blinded experiments?), and provide stronger rationales for the dose regimes (e.g. dose-response curves might be required) and the used late adolescent mice (P39-42) instead of adults mice.

3) To correctly interpret the fear conditioning data, it is critical to demonstrate whether PTZ-treated mice were able to learn during the training phase (data not shown) or whether PTZ treatment might induce hyperactivity, which could confound the results. Thus, fear conditioning training data need to be described and graph (e.g., including the 2 min of baseline activity to asses hyperactivity, the shock response, and the freezing acquisition to assess learning). In addition, it is unclear whether the reduction of freezing is an optimal measure of memory, since PTZ-treated rats might become hyperactive. A locomotor activity test would be important to address the possible confound of hyperactivity that could result in less freezing in the PTZ-treated rats. The results from figure 1C (left) may suggest that the PTZ+Veh rats are hyperactive since they froze significantly less than the PTZ+Wort during Pre CS conditions.

Additional comments:

- The authors emphasize that all effects are produced by a “single generalized seizure” but no continuous EEG recordings were performed to evaluate that. This important claim needs to be supported by data.

- Include the quantification of the PTZ-induced seizures in the result section (latency, scale, sex effects, etc.).

- The Wort dose does not appear to have completely blocked the increase in pS6/total S6 (Figure 1F). Could this be why freezing levels were still significantly lower than Sham +Veh (Figure 1C) when the rats were presented with the CS? Could this be why you were only able to partially rescue the short-term memory deficits? Another possibility you could address is that the Zhang and Wong 2012 paper administered Wort 30 minutes prior to PTZ seizures. Wort may have more impact if presented before the seizure.

- The variability (SEM) is quite high for the P-S6 levels of the Sham + Vehicle control group at 79.68. What is the sample size for the western blotting? Figure 1F for the control does not seem to have an error bar that reflects this variability.

- Wort did not appear to reduce basal levels of P-S6 or AKT as the Control+sham was not different from the Control+Wort groups. Is this what is normally found?

- For lines 224-226 for page 11 are the values correct for Sham + Wort compared to Sham + Veh animals? These values do not appear to reflect those in figure 1F.

- The figure legends are quite extensive and redundant with the results section. They need to be reduced.

Minor:

- Consider to use PI3K/AKT/mTOR pathway instead of I3K-mTOR pathway.

- Remove the “flash frozen” comment from the methods in the 6th page on line 91

- What was the decibel level of the white noise?

- If the results are shown in the graphs then it is not necessary to write the values into the results section.

- For line 164-166 for page 9 the statement was that there was not a statistically significant difference, yet a p value of p < 0.0001 was presented.

- Line 168 of page 8 change auditory memory to associative or cued conditioning deficits.

- On line 73 need to open the parenthesis for the end of 75 mg/kg.
